# Acetabular Revision with Structural Allograft and Cemented Acetabular Component: A Retrospective Case Series of 13 Patients

**DOI:** 10.1007/s43465-025-01683-0

**Published:** 2026-01-27

**Authors:** Maurício Rodrigues Miyasaki, Karen Barros Parron Fernandes, Vanderlei Montemor Bernardo, Eryleide Porfirio Ferreira, Fernando Takao Sassano Trigueiro Mendes, Carolina Morgato de Mello Miyasaki

**Affiliations:** 1 Irmandade da Santa Casa de Londrina, R. Espírito Santo, 523, Londrina, Paraná CEP 86038-350 Brazil; 2 Instituto de Ensino, Pesquisa e Inovação–Irmandade da Santa Casa de Londrina, Rua Souza Naves, 441, 14Th Floor, Room 141, Londrina, Paraná CEP 86010-510 Brazil; 3 Postgraduate programe of Health Science, Pontifícia Universidade Católica do Paraná, Paraná Curitiba, Brasil; 4 Département des Sciences de la Santé, Université du Québec à Chicoutimi, Québec, Canadá; 5 Universidade Federal Do Paraná, Curitiba, Paraná Brazil

**Keywords:** Revision total hip arthroplasty, Structural allograft, Cemented acetabular component, Acetabular defect, Bone stock restoration

## Abstract

**Background:**

Acetabular bone defects remain a challenging problem in revision total hip arthroplasty (THA). Structural allografts can restore the native hip center of rotation and replenish bone stock for potential future revisions, offering a cost-effective alternative in settings with limited access to porous metal augments. This study aimed to evaluate the short- to mid-term clinical and radiographic outcomes of acetabular revisions using structural homologous bone block grafts combined with cemented acetabular components.

**Methods:**

We retrospectively reviewed 13 consecutive patients (13 hips) with Paprosky type 2B, 2C, 3A, or 3B acetabular defects who underwent revision THA between February 2016 and October 2021. All cases were reconstructed using a structural homologous bone block graft, fixed to the ilium with screws, and a cemented polyethylene acetabular cup. Harris Hip Score (HHS) and standardized radiographs were assessed at the last follow-up. Implant survival and graft origin were analyzed using Kaplan–Meier curves and the log-rank test.

**Results:**

The mean patient age was 67.3 years (range, 44–85), and the mean follow-up was 54 months (range, 19–88). Two patients (15.4%) experienced reconstruction failure due to graft fragmentation and cup migration. The remaining 11 cases demonstrated satisfactory clinical and radiographic outcomes, with evidence of graft integration and no radiographic signs of loosening. Mean HHS improved from 34.5 ± 7.0 preoperatively to 70.1 ± 10.3 postoperatively.

**Conclusion:**

Structural allograft with a cemented acetabular component provided good short- to mid-term functional outcomes and radiographic stability in most patients. This technique remains a valuable, lower-cost option for managing major acetabular defects in resource-limited settings. **Level of evidence**: IV, retrospective case series.

## Introduction

Total hip arthroplasty (THA) is one of the most cost-effective surgical interventions in modern orthopaedics, with consistently high success rates [1]. Its widespread use, combined with an aging population and improved implant longevity, has led to an increasing number of primary THAs being performed in younger patients [2, 3]. Consequently, the likelihood of implant wear and failure over a patient’s lifetime has risen, driving an increased demand for revision THA procedures [3, 4].

Aseptic loosening remains one of the leading causes of THA failure [3]. This process is often insidious, with patients remaining asymptomatic until significant bone loss has occurred. Early detection through regular clinical and radiographic follow-up is essential to prevent the progression to advanced periprosthetic bone loss, which can necessitate complex reconstructions [5].

Ferreira et al. reported that, in 2010, Brazil had a total hip arthroplasty (THA) rate of 7.8 per 100,000 users of the public health system (SUS)—a dramatic deficit compared with the average of 191.8 per 100,000 inhabitants observed in European countries, the United States, and Australia [2]. This gap in healthcare provision may hinder adequate outpatient follow-up, delay the diagnosis of implant loosening, and potentially contribute to the occurrence of advanced cases of periprosthetic bone loss that require complex reconstructions.

Acetabular defects pose a major technical challenge in revision THA. Bone loss results from both the original surgical bone resection and periprosthetic osteolysis induced by polyethylene wear debris. These particles stimulate macrophage activation, leading to the release of pro-inflammatory cytokines such as interleukin-1 and tumor necrosis factor, which mediate bone resorption [6].

Accurate pre- and intraoperative classification of acetabular defects is critical for surgical planning. The Paprosky classification, introduced in 1997, remains one of the most widely adopted systems, stratifying defects based on ischial osteolysis, medial wall integrity, and superior migration of the hip center [7, 8].

Several strategies have been proposed for managing acetabular bone loss, including biological options such as impacted morselized bone grafts or structural allografts, and non-biological solutions such as trabecular metal augments, custom triflange components, or high hip center placement [8]. Structural allografts offer the advantage of restoring the native hip center of rotation while replenishing bone stock, which is particularly important in younger patients or in cases where future revision may be required.

In settings with limited access to advanced implants such as trabecular metal, structural allografts combined with cemented cups represent a cost-effective alternative. However, the long-term survival of such reconstructions remains a topic of debate in the literature. This study aimed to evaluate the clinical and radiographic outcomes of acetabular revision using monoblock structural homologous bone grafts and cemented acetabular components in patients with large acetabular defects.

## Materials and Methods

### Study Design and Patient Population

This retrospective case series study included 13 consecutive patients [13 hips] who underwent revision THA with acetabular reconstruction using a structural homologous bone block graft and a cemented polyethylene acetabular cup between February 2016 and October 2021 at our institution. All patients presented with major acetabular defects classified as Paprosky type 2B, 2C, 3A or 3B. Paprosky Type 3B defects were considered to include cases presenting with pelvic discontinuity.

The study was approved by the institutional Research Ethics Committee (BIOISCAL, protocol no. 6.432.307; CAAE: 70910823.3.0000). All procedures complied with the Declaration of Helsinki.

### Surgical Planning and Technique

Preoperative planning anticipated the use of structural bone grafts for Paprosky type 2B, 2C, 3A and 3B defects. Graphs from the femoral head, femoral condyle, or greater trochanter were employed, as per availability from the bone bank (Table 1).

**Table 1:** Distribution of cases

Patient	Age	Sex	Follow-up (months)	Paprosky	Graft origin	Radiographic failure	Final follow-up HHS score
1	44	M	88	3A	right femoral condyle	Yes	48
2	65	M	83	3B	right femoral condyle	Yes	48
3	75	M	78	3A	right femoral condyle	No	74
4	77	F	68	2B	right femoral condyle	No	75
5	56	F	68	2B	femoral head	No	75
6	78	M	65	2B	left femoral condyle	No	76
7	64	F	59	2B	right femoral condyle	No	78
8	85	M	54	3A	left femoral condyle	No	69
9	78	F	43	3A	left femoral condyle	No	78
10	55	F	31	2C	greater trochanter	No	68
11	76	F	28	3A	right femoral condyle	No	71
12	61	F	28	2B	femoral head	No	74
13	61	F	19	2C	femoral head	No	78

Distribution of cases according to age, sex, follow-up, acetabular defect classification according to Paprosky, graft used, and presence of radiographic signs of failure. Source: the Authors

**Fig. 1:**
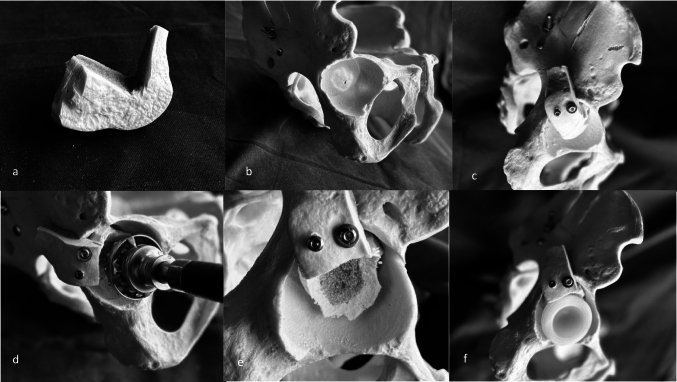
Steps of acetabular reconstruction: a. Lateral femoral condyle shaped to the acetabular defect; b. Acetabular defect; c. Structural graft fixed to the iliac bone; d. Reaming of the acetabulum and graft; e. Appearance of the reconstructed acetabular cavity; f. Positioning of the acetabular component supported on the graft

The graft blocks were carefully shaped to match the morphology of the acetabular defect and fixed to the ilium with 4.5-mm cancellous screws or 3.5-mm cortical screws. The constructs were subsequently reamed to recreate the native acetabular contour (Fig. 1). Morselized allograft was interposed at the acetabular floor and along the host–graft interface to enhance biological

All procedures were carried out through a posterolateral approach. After placement of the allograft, at least one 6-mm containment hole was drilled to ensure adequate purchase and passage through both the graft and the host bone. This hole facilitated optimal cement pressurization and mechanical interdigitation during subsequent cementation, after which a polyethylene cup was implanted. Femoral revision was also undertaken in every case, using either distal fixation techniques, cement-in-cement revision, or impaction grafting as appropriate

### Allograft Acquisition and Processing

All structural allografts utilized in this study were obtained from certified human bone banks: the National Institute of Traumatology and Orthopedics (Rio de Janeiro—RJ) or the Mackenzie Evangelic Hospital (Curitiba-PR).

Donor screening, tissue processing, and sterilization strictly adhered to the comprehensive standards established by the Brazilian National Health Surveillance Agency (ANVISA), ensuring quality and safety.

For operative logistics, grafts were handled under standardized conditions, transported in thermal packaging, and delivered to the operating room complex less than 24 h prior to the scheduled surgery, minimizing time outside controlled storage.

### Postoperative Management

In all cases, five to six intraoperative culture samples were collected, none of which showed bacterial growth. The standard antibiotic regimen consisted of cefazolin administered at anesthetic induction and continued at a dosage of 1 g every 8 hours until culture results were available. All patients were instructed to maintain partial weight-bearing for six weeks, progressing to full weight-bearing thereafter. No patient demonstrated acetabular construct failure during this period that required modification of postoperative management.

### Clinical and Radiographic Evaluation

All patients followed a standardized postoperative rehabilitation and follow-up protocol. Clinical and radiographic assessments were performed at the following intervals: 2 weeks (for suture removal and initial mobility guidance), 6 weeks (for radiographic assessment and progression to walking without crutches, as tolerated), 3 months, 6 months, and 1 year postoperatively. Following the first year, all patients were scheduled for annual follow-up appointments, always including a standardized radiographic series.

The Harris Hip Score (HHS) [9]was recorded at the last follow-up. Radiographic failure was strictly defined by the presence of one or both of the following criteria: [1] Component migration defined as any measured change in cup position exceeding 5 mm in any direction, as determined by comparing the latest follow-up radiograph to the immediate postoperative radiograph; or [2] The presence of progressive radiolucent lines measuring greater than 2 mm in width, involving any zone of the acetabulum, as assessed using the DeLee and Charnley classification system.

Radiographic assessment of graft incorporation and component migration was performed by a single, specialized orthopedic (The senior Author) who was blinded to the patient's final clinical outcome (Harris Hip Score) at the time of evaluation. Component migration and radiolucency were assessed using the criteria previously defined. Due to the study's design and limited cohort size, inter-observer reliability was not calculated.

### Statistical Analysis

**Fig. 2:**
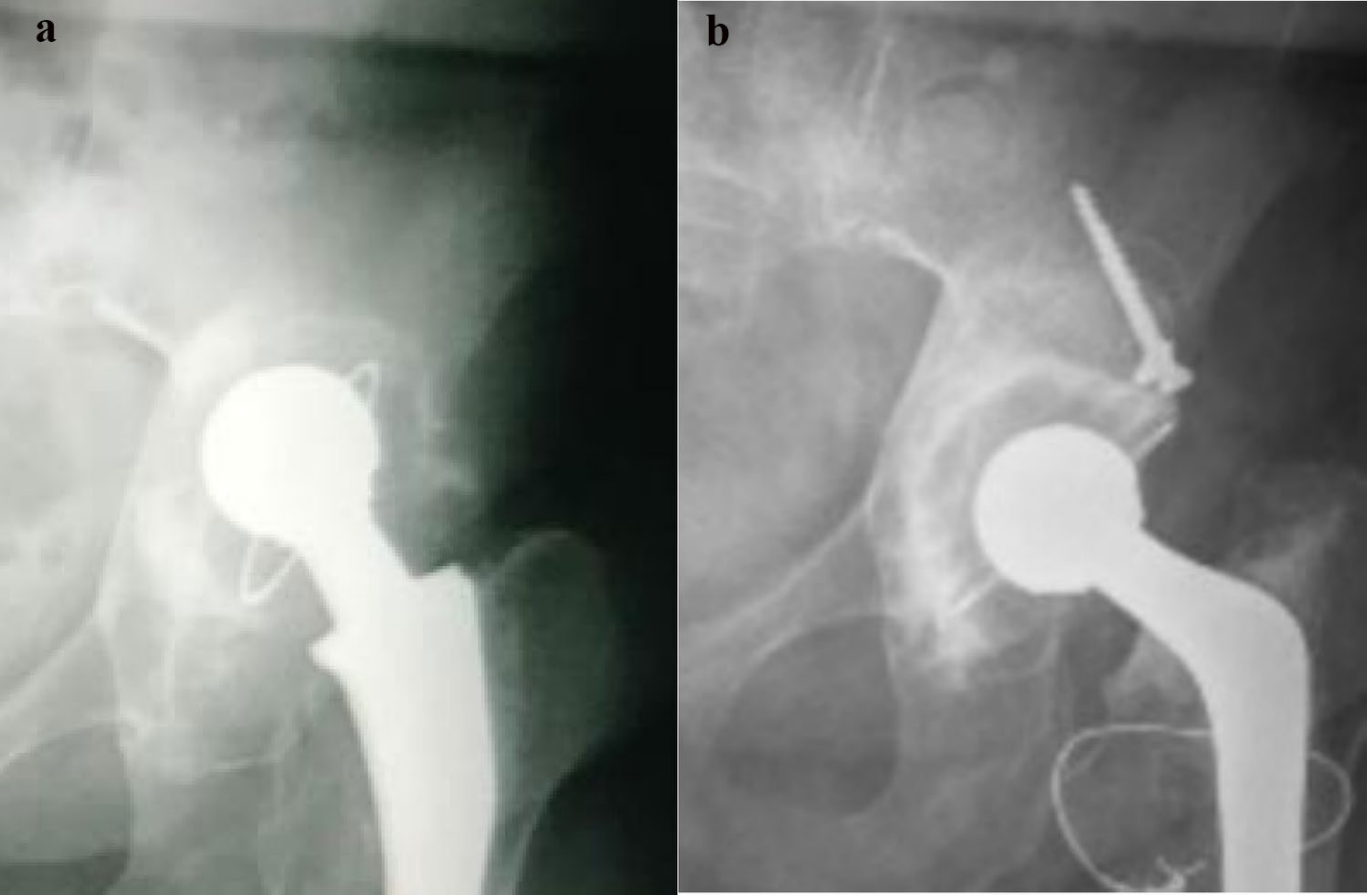
**A** Paprosky type 3A acetabular defect; **B** postoperative radiograph at 43 months follow-up. Source: the Authors

Data analysis used SPSS v18.0 (IBM). Kaplan–Meier curves and the log-rank test assessed survival differences by graft origin, with significance at *p* < 0.05 (Fig. 2).

## Results

### Demographics

Of the 13 patients, 8 were female and 5 were male. The mean age was 67.3 years (range, 44–85 years), and the follow-up period ranged from 19 to 88 months, with a mean of 54 months. All surgical procedures included in this series were performed between 2016 and 2020. The final data cut-off date for clinical and radiographic follow-up was May 2023. The demographic and clinical characteristics of the study population are summarized in Table 2. Acetabular defects were classified as Paprosky type 2B in 5 cases, type 2C in 2 cases, type 3A in 5 cases, and type 3B in 1 cases.

**Table 2:** Overall study population characteristics

Variable	Value
Age, mean ± SD (years)	67.31 ± 11.86
Gender	
Male, *n* (%)	5 (38.5)
Female, *n* (%)	8 (61.5)
Follow-up, mean ± SD (months)	54.77 ± 22.96
Preoperative HHS, mean ± SD	34.54 ± 7.03
Postoperative HHS, mean ± SD	70.15 ± 10.34
Clinical outcome	
Integration, *n* (%)	11 (84.6)
Failure, *n* (%)	2 (15.4)

Overall study population characteristics divided by age, gender, mean follow-up time, pre and postoperative Harris Hip Score and clinical features. Source: the authors

### Failure Analysis

Failure of the reconstruction was observed in two patients (15.4%), characterized radiographically by graft fragmentation and subsequent acetabular cup migration. These two failures were diagnosed early, occurring at **9 months** and **7 months** of follow-up, respectively.

The first failure involved a Paprosky type 3A defect, in whom the primary arthroplasty had been performed due to sequelae of an acetabular fracture. The second failure involved a Paprosky type 3B defect with pelvic discontinuity.

In both cases, the failures were determined to be mechanical/aseptic; there were no clinical or laboratory signs of infection noted during the several months of follow-up following the diagnosis of component loosening. At the time of this report, re-revision procedures had not yet been performed on these two patients.

### Survival Analysis

The overall construct survival rate at 5 years (60 months) was 84% [95% CI: 55%–95%]. The survival analysis (Kaplan–Meier) demonstrated a tendency toward lower construct survival in the subgroup utilizing the femoral condyle allograft. However, we emphasize that this descriptive finding must be interpreted with extreme caution due to the case series nature and the low number of observed failure events (*n* = 2), which severely limits statistical power. The most probable etiological factor for the early failures observed in these two patients lies in the extreme complexity and inherent instability of the defects (Paprosky 3A and 3B). The coincidence of both failures involving the femoral condyle allograft merely raises the hypothesis that this specific type of graft may have offered lower initial mechanical resistance in high-demand defects. This observation, while not inferential, suggests a need for further investigation to confirm whether graft origin is a true determinant of mechanical failure in complex acetabular reconstruction.

### Radiographic Findings

**Fig. 3:**
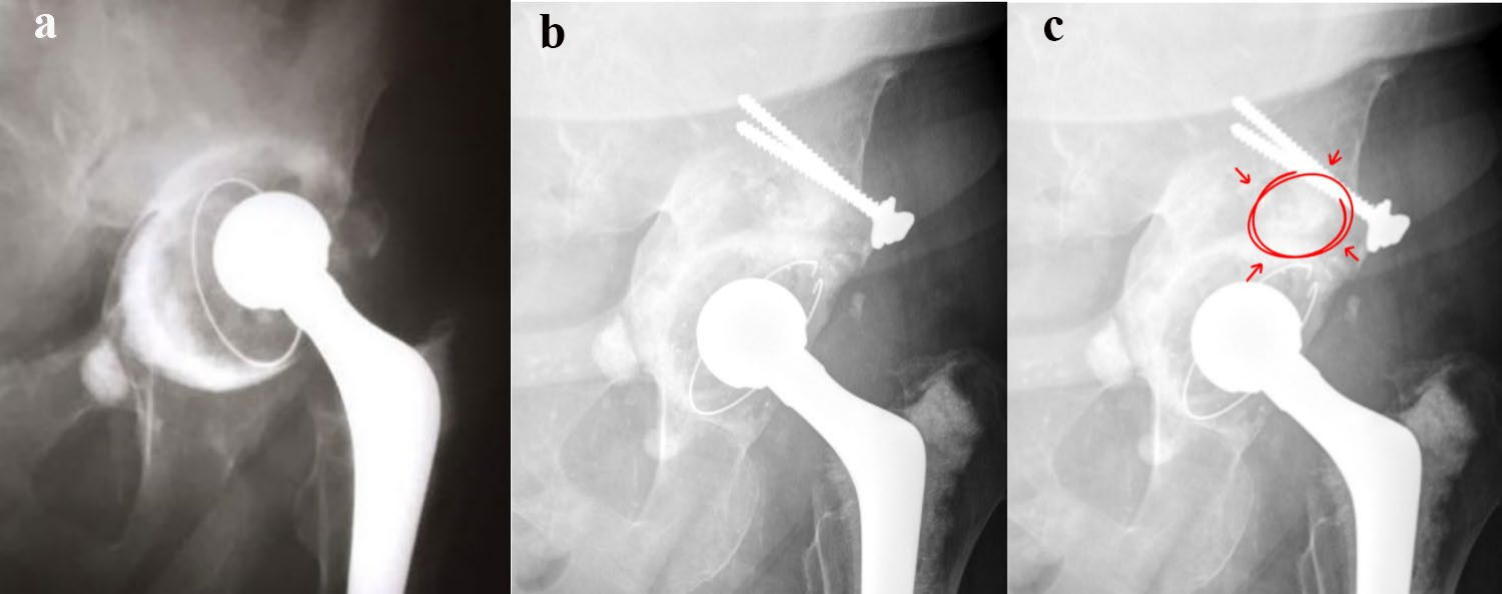
**A** Paprosky type 2B acetabular defect; **B** postoperative radiograph at 68 months follow-up. **C** Containment hole signalized in red. Source: the Authors

**Fig. 4:**
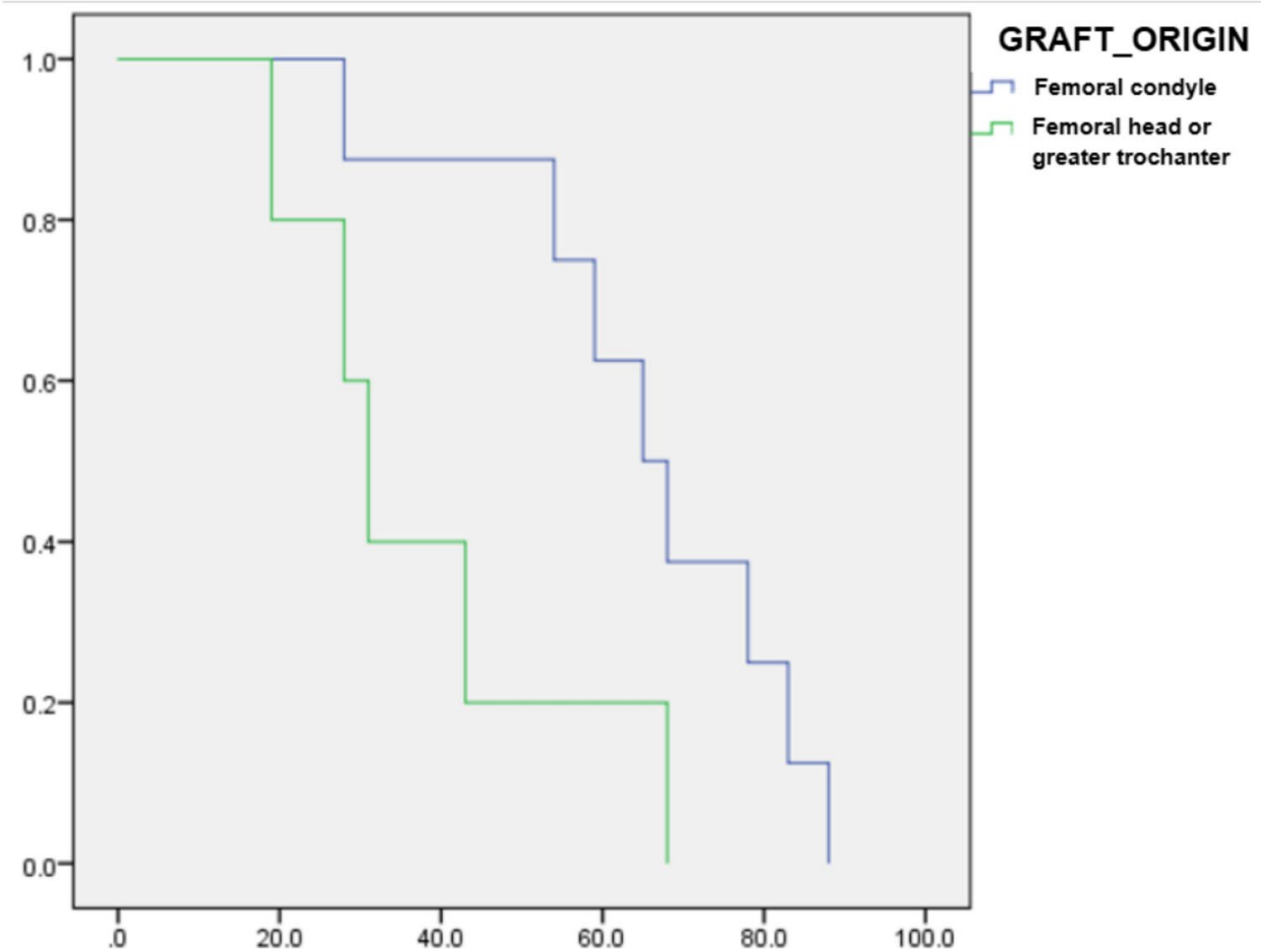
Kaplan–Meier cumulative survival curve showing association between graft origin and reconstruction failure; statistically significant difference (log-rank test, *p* = 0.04). Source: the Authors

One patient died during follow-up; her last evaluation was at 54 months, with no radiographic signs of graft failure and a Harris Hip Score (HHS) of 74. In the remaining 10 patients, radiographic evidence of graft consolidation was observed, with no findings suggestive of failure, such as screw breakage, radiolucent lines at the graft–host bone or cement–graft interface, or acetabular component migration (Figs. 3 and 4).

### Clinical Outcomes

Clinical outcome was defined by the functional improvement in the Harris Hip Score (HHS). The mean improvement in the HHS was 35.6 points (standard deviation, 12.49), with a 95% Confidence Interval (CI) ranging from 28.1 to 43.2 points. This improvement is considered highly clinically relevant, as the entire confidence interval lies substantially above the generally accepted thresholds for Minimal Clinically Important Difference (MCID). A good clinical result, defined a priori as an improvement of at least 20 points in the HHS, was achieved in all patients except the two who experienced reconstruction failure.

## Discussion

Revision total hip arthroplasty (THA) poses significant technical challenges, especially when major acetabular bone loss is present. Successful outcomes depend on accurate defect assessment and selection of a reconstruction strategy that restores stability and bone stock [8, 10, 11]. The use of bone grafting offers the advantage of restoring the anatomical hip center of rotation and replenishing bone stock, which is important for potential future revision surgeries [15]. In Brazil, the Public Health System (SUS) provides homologous bone grafts free of charge through certified bone banks [20], making this a relatively accessible option for complex acetabular reconstructions. In contrast, reconstruction techniques employing trabecular metal augments and cup-cage constructs have shown promising results [13]; however, their very high cost and the fact that these implants are not incorporated into the national healthcare system's list of covered materials (21) restrict their availability.

Bone grafts can serve either biological or mechanical purposes, depending on the clinical context [12]. Structural block grafts have less osteoinductive and osteoconductive capacity than impacted morselized grafts, but in our cases, they were employed to fill large acetabular defects and stabilize the implant. Furthermore, impacted allograft was used in all cases at the interface between the block graft and the host bone, as well as at the acetabular floor.

Some publications recommend using uncemented cups when structural bone allografts are employed, to achieve biological fixation in the portion of host bone in contact with the implant [6, 13]. Nevertheless, Ross et al. reported satisfactory long-term results with the use of block allografts and cemented cups [14]. There are also reports of excellent long-term outcomes with the use of structural autografts and cemented cups in cases of dysplasia [15].

The initial two cases in our series resulted in early mechanical failure. These patients presented with the highest degree of structural deficiency, involving a Paprosky type 3A defect secondary to complex fracture sequelae and a Paprosky type 3B defect with pelvic discontinuity. These scenarios represent the most challenging biomechanical environments in revision THA, characterized by high instability and load transfer challenges. The early failure observed is not merely a complication but the most critical lesson from this series. It demonstrates that the extent of bone loss and the lack of sufficient structural support were the primary determinants of outcome. The failure of the block allograft to withstand initial load in these Paprosky type 3 defects confirms that a constrained, load-sharing implant is mandatory to prevent early mechanical micromotion.

In light of this critical finding, we conclude that the use of reinforcement rings or cup-cage constructs is essential to provide immediate mechanical stabilization and effectively redistribute forces, thereby protecting the allograft during the critical phase of biological incorporation. Specialized reconstruction techniques, such as those employing trabecular metal augments and custom cup-cage constructs, consistently demonstrate superior long-term survival in Paprosky Type 3 defects, with survival rates often exceeding 90% at five years [16]. This reinforces that, while our technique successfully restores bone stock, the mechanical demands of the most severe defects require the immediate stability provided by these dedicated implant systems.

The descriptive survival analysis demonstrated an observed difference in construct survival based on graft origin (femoral condyle vs. other sources). However, we emphasize that this descriptive finding must be interpreted with extreme caution due to the case series nature and the low number of observed failure events (*n* = 2), which severely limits statistical power. The most probable etiological factor for the early failures observed in these two patients lies in the extreme complexity and inherent instability of the defects (Paprosky 3A and 3B). The coincidence of both failures involving the femoral condyle allograft merely raises the hypothesis that this specific type of graft may have offered lower initial mechanical resistance in high-demand defects. This observation, while not inferential, suggests a need for further investigation to confirm whether graft origin is a true determinant of mechanical failure in complex acetabular reconstruction.

Nevertheless, one of the major advantages of employing allografts in revision THA is their capacity to restore deficient bone stock, which has important implications not only for the immediate stability of the reconstruction but also for future procedures. Importantly, by replenishing bone stock, allografts provide surgeons with improved conditions for subsequent reconstructions, should further revisions become necessary, as the presence of restored bone facilitates implant fixation and enhances the range of reconstructive options available [17].

The remaining 11 patients demonstrated satisfactory clinical and radiographic outcomes at the time of evaluation. Although the mean follow-up of 54 months (range, 19–88 months) may be considered relatively short—given that the literature reports a higher incidence of structural allograft failure after 6 to 10 years [18–20]—the radiographic evidence of graft consolidation and maintenance of radiodensity provides an initial favorable outlook. However, we must interpret these outcomes with caution, recognizing that only long-term data will confirm the durability of the construct. Crucially, even if failure occurs in the long term, this technique provides a sustainable, cost-effective solution in resource-limited settings while serving the invaluable biological purpose of restoring bone stock, thereby simplifying and improving the foundation for any potential subsequent revision surgery. This dual benefit—providing a functional mid-term solution and facilitating future care—reinforces the relevance of this technique in diverse healthcare contexts.

The main limitations of this study must be critically addressed. First, the retrospective design introduces inherent risks of selection and information bias. Second, the small, heterogeneous cohort of 13 cases and the relatively short mean follow-up of 54 months significantly limit the statistical power and the generalizability of our findings. This constraint is particularly relevant given the low number of failure events (*n* = 2), which elevates the risk of a Type I error (false positive) in the survival analysis. Third, the variability in graft origin (femoral condyle vs. femoral head), although reflective of real-world practices, may have influenced incorporation and mechanical behavior without the ability to confirm this relationship statistically. Fourth, the absence of a dedicated control group treated with modern high-cost technologies (e.g., trabecular metal) hinders a direct comparison of outcomes. Finally, we acknowledge the limitation of relying solely on conventional radiography, as advanced imaging methods capable of detecting early micromigration or subtle signs of loosening were not employed. Despite these methodological constraints, the study provides valuable clinical insight into the application of a cost-effective technique, particularly within the context of the Brazilian public health system, where the structural allografting combined with cemented acetabular components stands out as a feasible and sustainable alternative for managing severe acetabular bone loss.

## Conclusion

Structural homologous bone block grafting combined with a cemented acetabular component showed encouraging short- to mid-term clinical and radiographic stability, representing a cost-effective and biologically sound alternative in resource-limited settings where advanced implants are not widely available. While failures in severe Paprosky type 3 defects suggest that additional reinforcement may be necessary, and the association between graft origin and failure warrants further study, these findings highlight structural allografting as a practical and sustainable option for acetabular reconstruction. Larger, multicenter studies with longer follow-up and comparative analyses are still needed to confirm its durability and better define its role in contemporary revision hip arthroplasty.

## Data Availability

The datasets generated and analyzed during the current study are available from the corresponding author on reasonable request.
